# Rice Defense Responses Orchestrated by Oral Bacteria of the Rice Striped Stem Borer, *Chilo suppressalis*

**DOI:** 10.1186/s12284-022-00617-w

**Published:** 2023-01-09

**Authors:** Rongrong Xue, Qing Li, Ruiqing Guo, Hui Yan, Xueyang Ju, Lu Liao, Rensen Zeng, Yuanyuan Song, Jie Wang

**Affiliations:** 1 Key Laboratory of Ministry of Education for Genetics, Breeding and Multiple Utilization of Crops, College of Agriculture, Fujian Agriculture and Forestry University, Jinshan, Fuzhou, 350002 China; 2 State Key Laboratory of Ecological Pest Control for Fujian and Taiwan Crops, College of Life Sciences, Fujian Agriculture and Forestry University, Fuzhou, 350002 China

**Keywords:** *Chilo suppressalis*, Oral secretion, Symbiotic bacteria, *Oryza sativa*, Induced defense

## Abstract

Plant defenses in response to chewing insects are generally regulated by jasmonic acid (JA) signaling pathway, whereas salicylic acid (SA) signaling is mainly involved in plant defense against biotrophic pathogens and piercing-sucking insects. Previous studies showed that both JA- and SA-related defenses in rice plants were triggered by the infestation of the rice striped stem borer (SSB, *Chilo suppressalis*), a destructive pest causing severe damage to rice production. Herbivore-associated microbes play an important role in modulating plant–insect interaction, and thus we speculate that the SSB symbiotic microbes acting as a hidden player may cause this anomalous result. The antibiotics (AB) treatment significantly depressed the performance of field-collected SSB larvae on rice plants, and reduced the quantities of bacteria around the wounds of rice stems compared to non-AB treatment. In response to mechanical wounding and oral secretions (OS) collected from non-AB treated larvae, rice plants exhibited lower levels of JA-regulated defenses, but higher levels of SA-regulated defenses compared to the treatment of OS from AB-treated larvae determined by using a combination of biochemical and molecular methods. Among seven culturable bacteria isolated from the OS of SSB larvae, *Enterobacter* and *Acinetobacter* contributed to the suppression of JA signaling-related defenses in rice plants*,* and axenic larvae reinoculated with these two strains displayed better performance on rice plants. Our findings demonstrate that SSB larvae exploit oral secreted bacteria to interfere with plant anti-herbivore defense and avoid fully activating the JA-regulated antiherbivore defenses of rice plants.

**Supplementary Information:**

The online version contains supplementary material available at 10.1186/s12284-022-00617-w.

## Key Message

- The incorporation of antibiotics to field-collected *Chilo suppressalis* larvae significantly reduce bacterial quantities and affect larval performance on rice plants.
- Field-collected *Chilo suppressalis* larvae deposit abundant bacteria onto feeding sites through oral secretion, which modulate defense responses in rice plants.
- Bacteria isolated from larval oral secretion (such as *Enterobacter and Acinetobacter*) contribute to preventing rice plants to fully activate jasmonic acid-mediated antiherbivore defenses.
- Our results indicate insect-associated bacteria play an important role for *C. suppressalis* to adapt to rice plants, which could also be treated as targets for developing novel pest management strategies.

**Supplementary Information:**

The online version contains supplementary material available at 10.1186/s12284-022-00617-w.

## Introduction

Most land plants generally respond to chewing insects by activation of jasmonic acid (JA)-mediated defenses, whereas they respond to biotrophic pathogens and some phloem feeding insects by elicitation of salicylic acid (SA)-regulated defense (Glazebrook 2005). Negative crosstalk between JA and SA signaling pathways has been reported in various plant species, which may allow plants to adjust defense responses to specific organisms (Beckers and Spoel 2006; Thaler et al. 2012). However, the magnitude of plant defense response could be modified by insect herbivores that exploit the antagonistic relationship between the JA-SA signaling pathways. Herbivores often exploit their symbiotic microbes to overwhelm plant-induced defenses by interfering the plant normal perception of herbivory (Sugio et al. 2015; Shikano et al. 2017).

Insects harbor diverse microbes that play a significant role in the nutrient supply, reproduction, digestion, pheromone synthesis, detoxification, etc. (Engel and Moran 2013; Douglas 2015). Besides, herbivorous insects-associated microbes can also manipulate plant induced defense responses directly or indirectly, leading to enhanced the adaptation of insects on host-plants (Zhu et al. 2014; Acevedo et al. 2015; Schmelz 2015; Mason et al. 2019a). The studies of insects co-opting symbiotic bacteria to suppress plant defense have been reported in several chewing insects, as some insects can orally secrete symbiotic bacteria that reside in the oral secretion (OS) during feeding (Chung et al. 2013; Acevedo et al. 2017; Wang et al. 2017; Yamasaki et al. 2021). For example, detection of bacteria deposited by Colorado potato beetle (*Leptinotarsa decemlineata*) leads to the enhancement of SA signaling-regulated defenses in tomato plants, and corresponding suppression of JA signaling-related defenses compared with those lacking symbiotic bacteria (Chung et al. 2013). Two bacterial isolates (*Pantoea ananatis* and *Enterobacteriaceae*-1) from the OS of fall armyworm (*Spodoptera frugiperda*) were found to suppress the activity of defensive enzymes in tomato plants and improve caterpillar growth (Acevedo et al. 2017). These studies suggest that suppression of plant defenses by insect symbiotic bacteria may be a widespread phenomenon allowing insect to adapt to host-plants.

Rice striped stem borer (SSB, *Chilo suppressalis*) is one of the destructive pests of rice (*Oryza sativa*) in Asia, southern Europe, and northern Africa (Jiang and Cheng 2003; Alfaro et al. 2009; Chen et al. 2011). The larvae cause severe yield and economic losses every year particularly in China due to the large cultivation of rice (Chen et al. 2011; He et al. 2013). SSB larvae bore into stem and feed inside, resulting in “dead hearts” at the tillering stage and “whitehead” at the reproduction stage (Pathak 1968). Rice plants infested by SSB larvae have higher levels of trypsin proteinase inhibitors, which is a direct defense compound that is generally regulated by JA signaling pathways (Zhou et al. 2009, 2011). Interestingly, a number of rice SA-related genes were also found to be up-regulated by SSB feeding (Liu et al. 2016). The mechanism responsible for this unusual induction of both JA and SA signaling pathway-regulated defenses by SSB feeding has not been well investigated. Here we hypothesized that insect-associated symbiotic microbes may trigger SA-related defense responses in rice plants during SSB feeding.

In this study, we tested whether microbes present in SSB larvae manipulated defense-related responses in rice plants. We initially compared the performance of SSB larvae treated with or without antibiotics (AB) on rice plants. Then, the presence of bacteria in larval OS and rice stem tissues damaged by larvae was determined by traditional plate culturing method and scanning electron microscopy observation. We also tested the effect of OS collected from larvae treated with or without AB on induced defenses in rice plants. Finally, culture-dependent method was used to identify bacteria present in the OS of SSB larvae, and the role of these bacterial isolates on plant defense responses was evaluated. These results will expand our understanding of the possible mechanism of SSB adapting rice defenses, and highlight the important role of insect-associated bacteria in shaping the complex interactions between insects and plants.

## Materials and Methods

### Plants

Rice (*Oryza sativa* cv. Nipponbare) seeds were surface-sterilized with 2% (v/v) sodium hypochlorite for 15 min and rinsed with distilled water three times. The sterilized seeds were transferred to seeding tray for germination. After seven days, the germinated seedlings were transplanted into a plastic box (length × width × height, 35 × 25 × 12 cm) containing 7 L nutrient solution prepared based on the receipt from International Rice Research Institute (Yoshida et al. 1976). The nutrient solution was changed every three days. All rice plants were grown for 40 days in a greenhouse at Fujian Agriculture and Forestry University (Fuzhou, China) with a day: night temperature regime of 28 °C (14 h): 22 °C (10 h), 70% relative humidity and a light intensity of 30,000 lx.

### Insect rearing

Field collected rice striped stem borer (*Chilo suppressalis*, SSB) larvae were collected from in a field located at the campus of Fujian Agriculture and Forestry University (Fuzhou, China, 119.23 N, 26.08 E). Laboratory-maintained SSB colony was kindly provided by Prof. Yunhe Li from the Institute of Plant Protection, Chinese Academy of Agricultural Sciences. The colony has been maintained in the laboratory for over 20 generations before being used for experiment. The egg masses of lab-reared insects were sterilized by soaking in 2.5% bleach solution for 5 min, and rinsed with distilled water three times. The sterilized eggs were transferred to petri dishes (diameter: 9 cm) for hatching. The neonates (10–15) were transferred into a glass tube (diameter: 2.4 cm; length: 7 cm) supplied with autoclaved artificial diet. The sterilized diet contained all components of a previously described diet (Han et al. 2012) but lacked bactericidal antibiotics that may compromise establishment of bacteria in subsequent experiments. When the larvae reached the third instar, the diet was changed and only 2–3 larvae were allowed in each tube to avoid cannibalization until pupation. The larvae were reared under conditions of 70–80% RH, 26 ± 1 °C, and a photoperiod of 16L: 8D. The pupae were removed from the rearing tubes and kept separately in petri dishes for adult emergence. The newly emerged adults were kept in cages containing rice plants at tillering stage for oviposition under conditions of 85–90% RH, 26 ± 1 °C, and a photoperiod of 16L: 8D. Then, the egg masses were collected, and the rice plants were replaced with fresh plants every day.

### Antibiotic treatment

To examine the effects of microbes in the oral secretion (OS) of SSB on mediating induced defense responses in rice plants, an antibiotic cocktail (AB) was used to reduce the microbes present in field-collected larvae as far as possible. The AB solution was made up of 0.01 g neomycin sulfate (MP Biomedicals), 0.05 g aureomycin (BioServ), and 0.003 g streptomycin (Sigma) dissolved in 50 ml of sterile MiliQ water as described by Chung et al. (2013). A tube of artificial diet (1 cm × 1 cm × 1 cm) was prepared and treated with 200 μL of AB, and placed in a laminar flow hood until dry. One larva at 3rd instar was placed in a plastic cup (1.5 oz) containing a tube of artificial diet with or without AB solution. Larvae were fed on artificial diet for 2 days, and then the body weight was measured. The AB-treated SSB larvae were used for the subsequent experiments. To verify the effects of AB treatment on removing insect-associated bacteria, we compared the bacterial quantities in the OS collected from SSB pretreated with or with AB. Two microliters of crude OS were freshly collected from SSB larvae treated with AB for 2 days by gently tapping larval heads according to a previously described procedure (Peiffer and Felton 2009; Acevedo et al. 2017), and diluted to 10^–3^ (μL) with sterilize water. The aliquot was then added to 2 × YT agar plate to count numbers of colonies (colony forming units, CFU), and each treatment had 5 replicates. The plates were incubated in a growth chamber at 27 °C for 24 h.

### Scanning electron microscope images

To verify that bacteria were secreted onto plant wounds during caterpillar feeding, SSB larvae with or without AB treatment were allowed to feed on rice stems for 1 d, and the damaged sections of stems were collected and prepared for scanning electron microscope (SEM) observation. Briefly, the SSB larvae were fixed on the rice stem using the 75% ethanol sterilized plastic tubes and autoclaved cotton according to the methods described by Jiao et al. (2018). After 1 h of feeding, the stems with visible injury caused by both AB-treated larvae and larvae with AB treatment were collected and placed in a centrifuge tube containing formalin fixing solution (Solarbio, Beijing). The samples were stored at 4 °C until being dehydrated and coated with gold. The images were captured using SEM (JSM-6380LV) located at Fujian Academy of Agricultural Sciences (Fuzhou, China).

### Effect of antibiotic treatment on larval performance on rice plants

To examine whether AB treatment affect the performance of SSB larvae on rice plants, 2-d-old 3rd-instar larvae with or without AB treatment were fixed on the stems of 40-d-old rice plants using a plastic tube (diameter: 3 cm, length: 6 cm) with both ends plugged with cotton as described above. Thirty larvae with similar size (20 ± 5 mg) from both AB treatment and non-AB treatment were selected and allowed to feed on the stem. After 3 days of feeding, the percentage of successful penetration was determined as the number of larvae successfully penetrating divided by the total number of larvae inoculated (Hou and Han 2010). Then, the larvae were removed, and the mass of larvae that had successfully bored into stems were weighted. The experiments were repeated three times, and each treatment had three replicates.

### Effect of OS from AB-treated larvae on defense responses in rice

Oral secretions were collected from both AB-treated larvae and non-AB treated larvae, and diluted 1:9 v/v with sterile MilliQ water. Each rice plant was mechanically wounded using a puncher to punch a 0.5 cm diameter hole in the main stem and 20 μL of diluted OS were applied the wounded sites. Control plants were wounded and treated with 20 μL of sterile water. The leaf tissue around the feeding sites was harvested 24 h later for gene expression analysis. After 48 h, plant tissues around damaged sites were collected for assessing the activities of two key defensive enzyme in rice analysis. The gene transcription in rice plants happens intensively in 24 h after damage occurs (Ye et al. 2012), while induced anti-herbivore enzymes in plants peak at approximately 48 h after the infestation (Constabel et al. 1995; Lin et al. 2022). For insect bioassay, the remaining tissue from each damaged leaf was used to SSB larval growth. All 3rd-instar larvae were allowed to feed on rice stems with different treatment for 3 d, and then the mass gain of larvae on each plant were weighted. Each treatment had more than 30 biological replicates, and the experiment was repeated three times.

### Analysis of LOX activities

Lipoxygenase (LOX) catalyzes the initial reaction in JA biosynthesis pathway (Stenzel et al. 2003). In rice plants, LOX could be significantly activated at 48 h after infestated by chewing insect such as rice leaf folder (*Cnaphalocrocis medinalis*) (Ye et al. 2012). LOX activity was measured as conjugated diene formation with slight modification (Macri et al. 1994). Rice stem samples (0.1 g) were ground in liquid nitrogen and extracted with 1 mL of ice-cold 0.5 M Tris–HCl buffer (pH 7.6) and centrifuged at 12,000 g for 20 min at 4 °C. The supernatant was transferred into a new centrifuge tube and kept at 4 °C until used. The substrate contained 0.5% (v/v) Tween20 and 1.6-mM linoleic acid in 0.1 M PBS (pH 7.6). The reaction was initiated by adding 0.2 mL of supernatant in 4.8 mL of the substrate. An extinction coefficient of 25 mM^−1^ cm^−1^ was used to convert absorbance values at 234 nm to nmol of conjugated diene.

### Quantification of PI contents

Trypsin protease inhibitor (PI) contents were measured using enzyme linked immunosorbent assay (ELISA) kits (Shanghai Enzyme-linked Biotechnology Co., Ltd., Shanghai, China). The stem samples were ground into a fine power in liquid nitrogen using a mortar and pestle, and each sample (0.1 g) was homogenized in 1 mL of 0.01 M Phosphate Buffered Saline (PBS) buffer (pH = 7.4). Samples were centrifuged at 5000 × g for 10 min at 4 °C, and the supernatant was collected into a new 1.5-mL centrifuge tube. The ELISA experiments were performed following the protocols provided with the kits. The optical density values were recorded at 450 nm using a microplate spectrophotometer (BioTek, Vermont, USA). The protein concentrations in plant samples were measured using a bicinchoninic acid (BCA) protein assay kit (Aidlab Biotechnologies Co., Ltd., Beijing, China) according to the manufacturer’s instructions. The amount of protease inhibitor was calculated based on a standard curve, and results were determined as µg protease inhibitor per mg protein.

### Analysis of gene expression by quantitative real-time PCR

Quantitative real time PCR was used to examine the expression levels of different genes related with defense responses in rice plants. RNA extraction was referred to CWBIO’s Ultrapure RNA kit (ComWin Biotech, Beijing, CW0581M) manufacturers protocol. Briefly, 50 mg of plant tissue was mixed with 1 ml of TRLzon Reagent (ComWin Biotech, Beijing), and allowed to stand at room temperature for 5 min. Chloroform (200 μl) was added, and vigorously shaken for 15 s at room temperature for 2 min, and then centrifuged at 4 °C and 12,000 rpm for 10 min. The pre-extracted RNA was transferred to a new RNase-free tube, an equal volume of 70% ethanol was added, inverted and mixed. RNA washing solution was added to the centrifuge in order, the obtained RNA was stored at − 70 °C. Total RNA (1 µg) was then pipetted for cDNA synthesis using the GoScript TM Reverse Transcription System (Promega Biotech, Beijing). Real-time PCR was performed according to the procedure of Ultra SYBR three-step fluorescence quantitative PCR kit (ComWin Biotech, Beijing). Reaction conditions for thermal cycling were 95 °C for 10 min, followed by 40 cycles of 95 °C for 10 s, 60 °C for 30 s, then 72 °C for 32 s. Fluorescence data were collected during the cycle at 60 °C. The relative transcript levels of the target genes in samples were determined according to the standard curve. A rice actin gene *OsActin* was used as an internal standard to normalize cDNA concentration. The primers used for qRT-PCR for all tested genes (JA-related genes: *OsAOS2* and *OsLOX*; SA-related genes: *OsPAL1* and *OsPR-1a*) are listed in Additional file 1: Table S1.

### Classification of bacteria in larval oral secretion

To isolate bacteria in OS from SSB larvae, the OS was collected from non-AB-treated larvae and cultured on LB agar plates. Single colonies were randomly selected and purified and cultured on BPA medium. A single colony was collected with a 20 µl pipette and transferred to a PCR tube containing 10 µl of sterile water. DNA was obtained by cleavage at 95 °C for 10 min. The next step was PCR amplification. The PCR reaction contained 1 µl of 10 µM 16S rRNA primer (530 F 5′-GTG CCA GCM GCC GCG G-3′ and 1392R 5′-ACG GGC GGT GTG TRC-3′), 12.5 µL of the GoTaq Green Master Mix (Promega), 2 µL of the bacteria DNA, and 8.5 µL of MQ water for a total volume of 25 µL. The PCR conditions as follows: 95 °C for 5 min, followed by 30 cycles of 95 °C for 1 min, 53 °C for 1 min, and 72 °C for 1 min 30 s, and finally 72 °C for 7 min. The PCR production was sent to BioSune bio-company (Fuzhou, China) for sequencing, the 16S rRNA sequences were analyzed using BLAST the nucleotide database of the National Center for Biotechnology Information (NCBI) with sequence similarity greater than 95%.

### Effect of oral secreted bacteria on plant induced defenses

The effect of SSB larval OS-associated bacteria on plant defense responses was examined. To determine whether bacterial isolates cultured from SSB larvae oral secretion suppressed plant defenses, rice stems mechanically wounded by a puncher were inoculated with individual bacterial isolate (OD600 = 0.1. c.a. 10^7^ CFU/mL) collected from SSB larval OS and cultured on 2 × YT liquid media. After 48 h, stem tissue around the wounded site was harvested to analyze LOX activity. The impact of individual isolated bacterium on plant defense responses was compared with rice plants treated with wounding and application of liquid media. Leaf samples were frozen in liquid nitrogen and stored at − 80 °C until analysis.

### Generation of axenic and gnotobiotic SSB larvae

Axenic and gnotobiotic SSB larvae were produced by following the methods described by Mason et al (2019b) with a small modification. All the processes of axenic rearing were conducted in a laminar flow hood. The SSB eggs were sterilized as described above, and the larvae were maintained on autoclaved diet. To generate gnotobiotic larvae from these axenic larvae, bacteria were administered to axenic larvae through inoculation of artificial diet. Liquid cultures of both *Enterobacter* and *Acinetobacter* were freshly grown overnight on 2YT media (16 g/L tryptone; 10 g/L yeast extract; 5 g/L NaCl). Cells were collected and re-suspended in 10 mM of sterilized MgCl_2_ solution. Each group of insects (5 larvae) was provided with approximate 10^7^ cells, while control insects received an identical volume of MgCl_2_ solution. Insects were allowed to feed on diet 1-cm diameter diet cube inoculated with individual bacterial isolate UV sterilized diet cups for 2 days before using in the subsequent experiments.

### Detection of bacteria from gnotobiotic larvae deposited onto plants

To determine whether gnotobiotic larvae secreted the same bacteria onto damaged plants, surface-sterilized gnotobiotic larvae were placed on the rice stems confined by sterilized tubes, and undamaged plants were treated with an empty tube. The rice stems were wiped with 70% ethanol before the infestation of SSB larvae. After 24 h of feeding, the tubes were removed, and the boring sites on the stems were detached by sterilized scissors. The collected tissues were suspended in 2.5 mL tube with 1 mL liquid 2 × YT media and cultured overnight in a rotary shaker at 200 rpm at 27 °C. Ten microliters of the culture were heated at 95 °C for 10 min. DNA released from the bacterial cells was amplified with specific primers showed in Additional file 1: Table S1. Gel electrophoresis was conducted to examine the PCR products, which were then sequenced and verified.

### Effect of OS from gnotobiotic larvae on defense responses in rice

Twenty microliters of OS collected from larvae inoculated with *Enterobacter* were applied to the wounded sites of rice stems using a puncher. Plant tissue around the damaged area was harvested for measuring the expression levels of plant defense signaling pathway-related genes (24 h later) and LOX activity (48 h later) according the detailed methods described above. For insect bioassay, 3rd instar larvae were allowed to feed on plants pre-treated with OS collected from axenic or gnotobiotic larvae. After 3 days of feeding, the mass gain of larvae on each plant was weighted. Each treatment had more than 30 replicates, and the experiment was repeated three times.

### Statistical analysis

The normal distribution and homogeneity of all data sets were verified before analysis. Minitab 16 (Minitab Inc., State College, USA) was used for data analysis. Data were analyzed by one-way analysis of variance (ANOVA) followed by Fisher’s test or unpaired student’s *t*-test (significance level, *p* < 0.05). The sample size and number of replicates for all the assays and analysis are indicated in the legends of the corresponding figures. All figures were generated using GraphPad Prism 8 (GraphPad Software Inc., San Diego, CA, United States).

## Results

### SSB larvae fed with antibiotics show poorer performance on rice plants

**Fig. 1:**
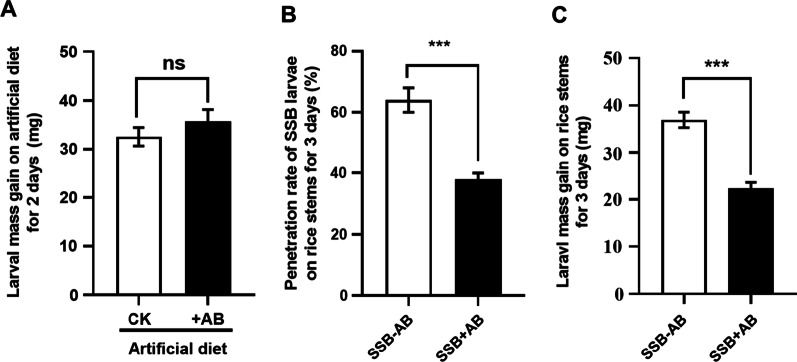
Antibiotic (AB) treatment suppressed the penetration ability and growth of *Chilo suppressalis* (SSB) larvae on rice plants. **A** The mass gain of field-collected SSB larvae at 3rd instar was measured after 2 days of feeding on artificial diets with or without AB (Unpaired *t*-test; *t* = 1.021, df = 38, *P* = 0.3137, n = 20). CK, artificial diet without adding AB; + AB, artificial diet contained AB. **B** The penetration rate of field collected SSB larvae with or without AB treatment within 3 days of feeding on rice plants (Unpaired *t*-test; *t* = 5.814, df = 8, *P* = 0.0004, n = 5). **C** The mass gain of field-collected SSB larvae with or without AB treatment that had successfully bored inside rice stems (Unpaired *t*-test; *t* = 6.974, df = 28, *P* < 0.001, n = 15). Values are means ± SEM, and asterisks indicate significant differences between treatments (*n.s.*, not significant; ***, *P* < 0.001). SSB-AB, field-collected SSB larvae without AB treatment; SSB + AB, field-collected SSB larvae with AB treatment

To examine whether antibiotics (AB) treatment affects the performance of field-collected SSB larvae on rice plants, 3rd instar larvae were firstly allowed to fed on artificial diet with AB amendment for 2 days and then transferred to the rice plants. When larvae fed on artificial diet, there was no significant difference in larval mass gain of SSB treated with or without AB (Fig. 1A; *t* = 1.021, *P* = 0.3137 > 0.05). Interestingly, we observed that larvae without AB treatment show higher boring rate than AB-treated larvae when fed on rice plants (Fig. 1B; *t* = 5.814, *P* = 0.0004). In addition, we randomly selected 15 larvae that had fully bored into rice stem from each treatment, and found the larvae without AB treatment gained more mass than AB-treated larvae when fed on rice stem for 3 days (Fig. 1C; *t* = 6.974, *P* < 0.001). These results suggested that SSB larvae pre-treated with AB showed poorer performance on rice plants compared to larvae without AB treatment.

### Antibiotics reduce bacterial number in the oral secretion of SSB

**Fig. 2:**
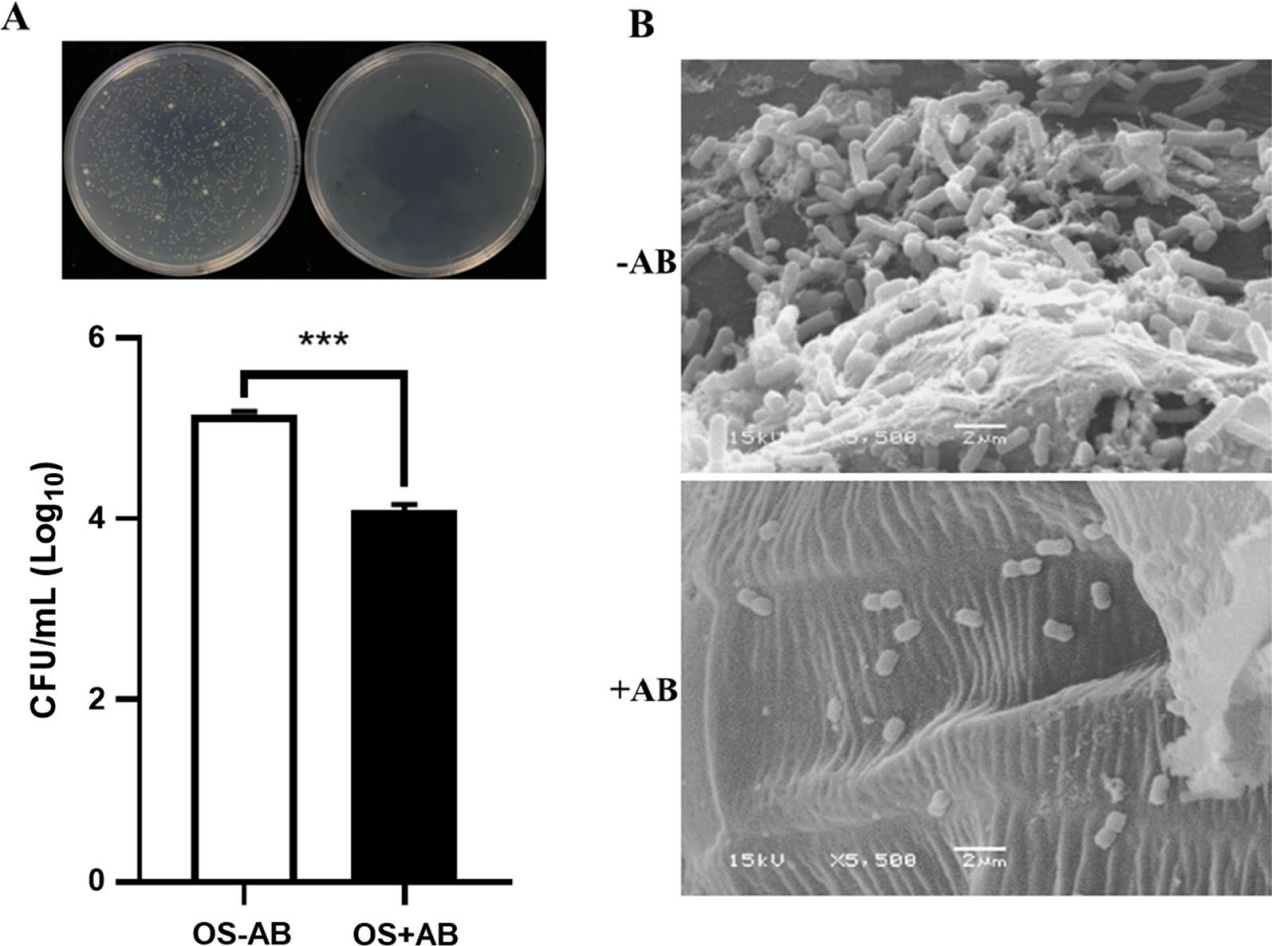
Field-collected *Chilo suppressalis* (SSB) larvae with AB treatment orally secreted lower quantities of bacteria onto the rice stems around the larval feeding area. **A** Comparison of bacteria quantities in the oral secretion (OS) of field-collected SSB larvae with 2 days of feeding on artificial diets with or without AB. Values are log_10_ transformed means ± SEM (n = 5), and asterisks indicate significant differences between treatments (Unpaired *t*-test; *t* = 14.88, df = 8, *P* < 0.001). **B** Scanning electron microscopy images of bacteria secreted onto rice stems during 1 h of feeding by SSB larvae with or without AB treatment (Scale bar = 2 μm)

Oral secretion (OS) collected from field-collected larvae fed on artificial diet inoculated with or with AB treatment were cultured for quantifying the number of bacteria, and the result showed that AB treatment significantly reduced the number of bacteria presented in the OS with a 10- to 15-fold reduction compared to the larvae without AB treatment (Fig. 2A; Unpaired *t*-test; *t* = 14.88, *P* < 0.001). In addition, both AB-treated larvae and non-AB-treated larvae were allowed to feed on rice stem for 1 h, and the scanning electron microscopy (SEM) observation showed that AB-treated larvae deposited much fewer bacteria on the feeding sites compared to the number of bacteria deposited by non-AB treated larvae (Fig. 2B). These results demonstrated that SSB larvae orally secreted detectable number of bacteria onto the feeding sites of host plants and the antibiotic treatment was effective in removing the number of bacteria presented in OS of SSB larvae.

### Symbiotic bacteria in larval oral secretion suppress plant defense responses

**Fig. 3:**
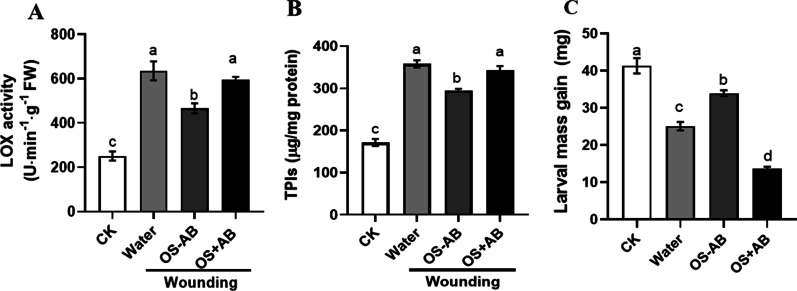
Defensive responses of rice plants in response to oral secretion (OS) collected from *Chilo suppressalis* (SSB) larvae with or without AB treatment. Rice stem samples were harvested 48 h after mechanical wounding with application of OS + AB and OS-AB, and the Lipoxygenase (LOX) activity (**A**) and Trypsin protease inhibitor (PI) content (**B**) were measured (LOX, *F*_(3,36)_ = 40.39, *P* < 0.001; PI, *F*_(3,36)_ = 106.5, *P* < 0.001; n = 10). CK, untreated rice plants; Water, rice stems wounded and applied with sterilized water; OS-AB, rice stems wounded and treated with oral secretion collected from larvae without AB; OS + AB, rice stems wounded and treated with oral secretion collected from larvae with AB. **C** The 3rd instar larvae were allowed to feed on rice plants pre-treated by mechanical wounding with application of OS + AB and OS-AB for 3 days, and then larval mass gain was determined (*F*_(3,36)_ = 106.5, *P* < 0.001; n = 10). CK, larvae fed on untreated rice plants. Values are means ± SEM. Different letters indicate statistically significant differences between treatments based on an ANOVA followed by LSD test (*P* < 0.05)

To investigate whether bacteria present in oral secretion (OS) affect plant induced defenses, OS collected from AB-treated or untreated larvae was applied to mechanically wounded rice plants, and then plant defense-related enzyme activities were measured. Wounded plants treated with OS collected from larvae without AB treatment showed lower levels of LOX (Fig. 3A; *F*_(3,36)_ = 40.39, *P* < 0.001) and TPI (Fig. 3B; *F*_(3,36)_ = 106.5, *P* < 0.001) than those wounded and treated with OS collected AB-treated larvae. The newly molted larvae of 3rd instar that fed on rice stem treated with OS collected from larvae without AB treatment for 3 days gained more weight than those that fed on rice stem damaged by OS collected from AB-treated larvae (Fig. 3C; *F*_(3,36)_ = 106.5, *P* < 0.001).

**Fig. 4:**
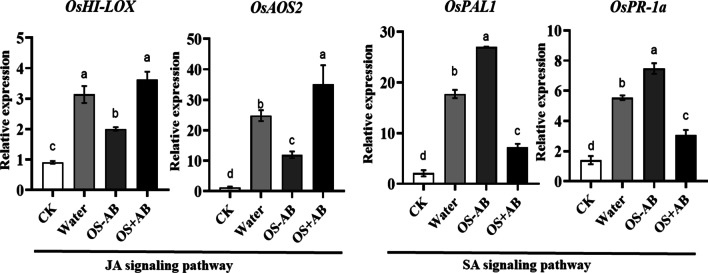
Expression levels of JA and SA signaling pathway-related genes in rice plants damaged by oral secretion collected from AB-treated or untreated larvae. Gene expression was analyzed 24 h after treatment. Different letters above bars indicate statistically significant differences between treatments (ANOVA, *P* < 0.05; followed by LSD test; *OsHI-LOX*, *F*_(3,20)_ = 39.44, *P* < 0.001; *OsAOS2*, *F*_(3,20)_ = 19.78, *P* < 0.001; *OsPAL1*, *F*_(3,20)_ = 332, *P* < 0.001; *OsPR-1a*, *F*_(3,20)_ = 85.85, *P* < 0.001; n = 6). CK, untreated rice plants; Water, rice stems wounded and applied with sterilized water; OS-AB, rice stems wounded and treated with oral secretion collected from larvae without AB; OS + AB, rice stems wounded and treated with oral secretion collected from larvae with AB

The *OsHI-LOX* and *OsAOS2* were selected as JA-marker genes because they play a key role in JA synthesis pathway. For SA-marked genes, *OsPAL1* and *OsPR-1a* were selected. Treatment by OS collected from untreated larvae decreased expression levels of the JA-responsive *OsHI-LOX* and *OsAOS2* compared with damaging by AB-treated larvae, whereas damaging by untreated larvae increased the expression of SA-responsive *OsPAL1* and *OsPR-1a* (Fig. 4). Taken together, these results demonstrated the microbes in OS from SSB larvae suppressed the antiherbivore defense responses in rice plants.

### Culture and identification of bacteria isolated from the OS of SSB

Since antibiotic treatment significantly affects larval performance and the components in larval oral secretion played a key role in mediating plant defense, it was speculated that symbiotic bacteria presented in the oral secretion may cause the fluctuation. To obtain the specific bacteria and further investigate the function of insect symbiotic bacteria in regulating the relationship of plant–insect, traditional culturing method was used to obtain the culturable bacteria in OS of SSB larvae. Seven bacterial isolates were isolated from OS of field-collected SSB larvae. Sequencing of the 16S rRNA region of all bacterial isolates using primers 27F and 1492R showed that these bacteria from OS shared high similarity (> 97%) with genus *Acinetobacter*, *Lactococcus*, *Aeromonas*, *Chryseobacterium*, *Brucella*, *Klebsiella*, and *Enterobacter* (Table 1). The sequence data of all the bacterial isolates has been submitted to GenBank with accession numbers from MZ424720 to MZ424726. These isolates were used for further functional characterization in the following experiments.

**Table 1:** Identification of bacteria in the OS of field-collected SSB

Isolate	Closest genus	GenBank Accession
S1	*Acinetobacter soli*	MT081629.1
S2	*Lactococcus garvieae*	NR_113268.1
S3	*Aeromonas media*	NR_119041.1
S4	*Chryseobacterium cucumeris*	NR_156145.1
S5	*Brucella pseudogrignonensis*	NR_042589.1
S6	*Klebsiella pneumoniae*	NR_117684.1
S7	*Enterobacter asburiae*	NR_024640.1

### Identification of bacterial isolates suppressing plant defenses

**Fig. 5:**
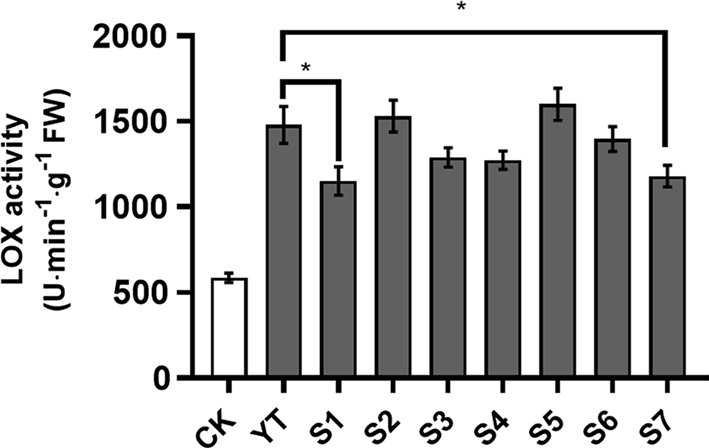
LOX activities in rice plants treated with mechanical wounding and individual bacterial strains isolated from oral secretion of SSB larvae. Values are means ± SEM (n = 10). CK, untreated rice plants. Asterisks indicate significant differences between YT media treatment and individual bacterial isolates treatment (unpaired *t*-test; YT vs. S1 (*Acinetobacter*), *t* = 2.395, df = 18, *P* = 0.0277; YT vs. S7 (*Enterobacter*), *t* = 7.967, df = 18, *P* < 0.001)

To investigate whether individual bacterium isolated from OS affect plant induced defenses, individual bacterial isolate was directly applied to mechanically wounded rice plants to measure the plant defense-related enzyme activities. The result showed that 2 out of 7 bacterial isolates (*Enterobacter* and *Acinetobacter*) significantly decreased LOX activity in rice plants compared with plants treated with 2 × YT media (Fig. 5; YT vs. *Enterobacter*, *t* = 7.967, *P* < 0.001; YT vs. *Acinetobacter*, *t* = 2.395, *P* = 0.0277). These results demonstrated symbiotic microbes present in the OS of SSB larvae suppressed rice induced defense.

### Reintroduction of OS-harbored bacteria to SSB larvae suppressed plant defenses

**Fig. 6:**
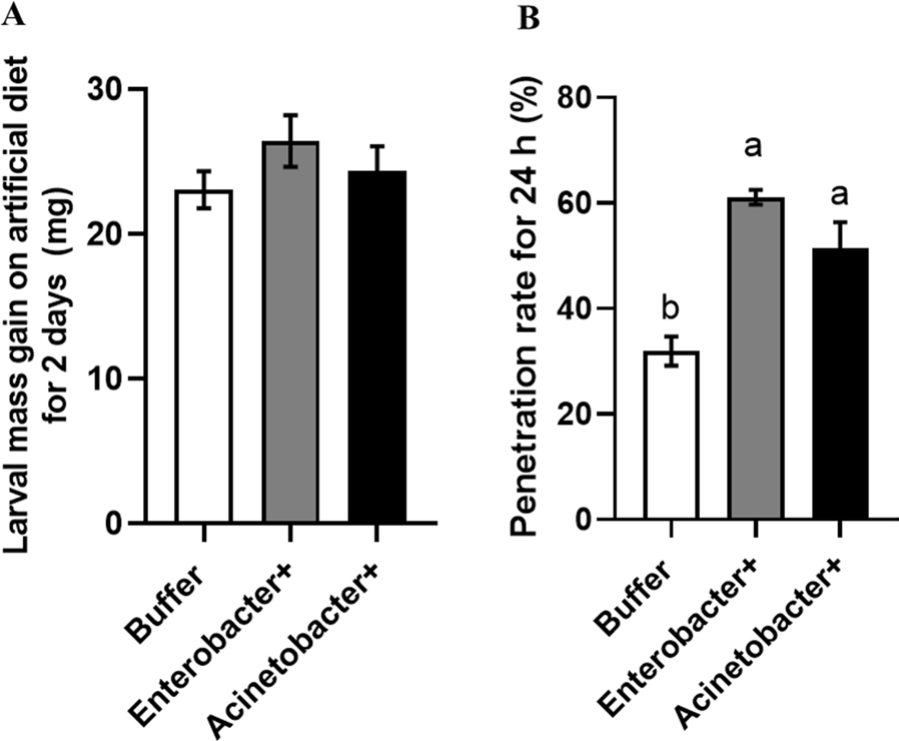
Impacts of bacterial inoculation on the performance of SSB larvae. Axenically reared insects were inoculated with 2 bacterial isolates on artificial diet for 2 days and transferred to rice plants. **A** Growth of SSB larvae were analyzed when fed on artificial diet inoculated with *Enterobacter* and *Acinetobacter* (*F*_(2,100)_ = 1.118, *P* = 0.3309; n = 34–35). **B** The penetration rate of SSB larvae inoculated with *Enterobacter* and *Acinetobacter* within 3 days of feeding on rice plants were measured (*F*_(2, 6)_ = 19.06, *P* = 0.0025; n = 3). Values are means ± SEM. Different letters above bars indicate statistically significant differences between treatments based on an ANOVA followed by LSD test (*P* < 0.05). Buffer, SSB larvae inoculated with 10 mM of MgCl_2_ solution; *Enterobacter* + , SSB larvae inoculated with cells of *Enterobacter*; *Acinetobacter*+, SSB larvae inoculated with cells of *Acinetobacter*

**Fig. 7:**
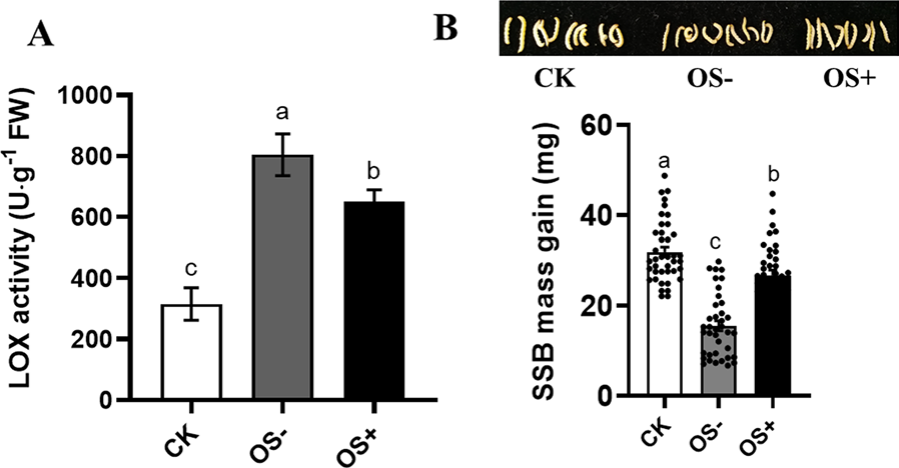
Defensive responses of rice plants in response to oral secretion (OS) collected from *Chilo suppressalis* (SSB) larvae inoculated with *Enterobacter*. (A) Rice stem samples were harvested 48 h after mechanical wounding with application of OS collected from *Enterobacter* inoculated SSB larvae (OS+ *Enterobacter*) and OS collected from axenic larvae (OS−), and the levels of defense-related LOX enzymes were measured (*F*_(2,42)_ = 20.43, *P* < 0.001, n = 15). CK, untreated rice plants. **B** The 3rd instar larvae were allowed to feed on rice plants pre-treated by mechanical wounding with application of OS+ *Enterobacter* and OS- for 3 days, and then larval mass gain was determined (*F*_(2,113)_ = 54.83, *P* < 0.001; n = 38–40). CK, SSB larvae fed on untreated rice plants. Values are means ± SEM. Different letters above bars indicate statistically significant differences between treatments based on an ANOVA followed by LSD test (*P* < 0.05)

**Fig. 8:**
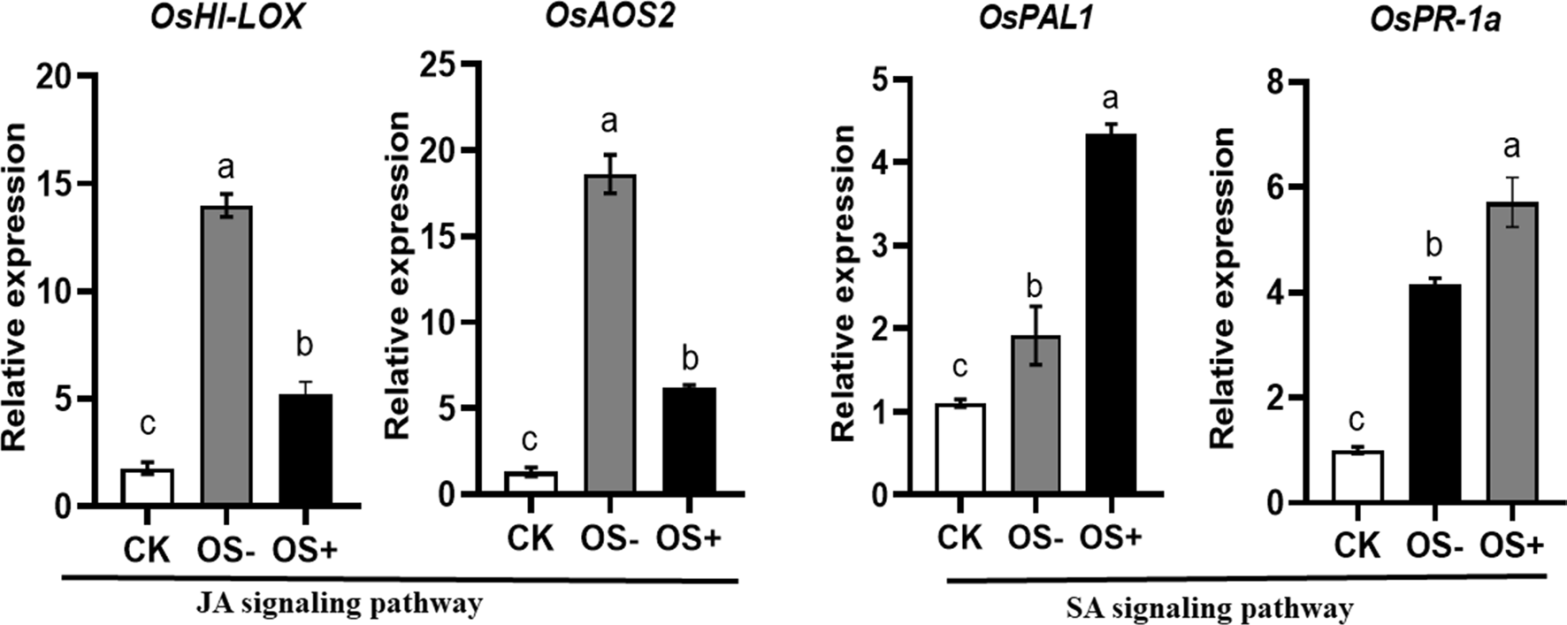
Expression levels of JA and SA signaling pathway-related genes in rice plants treated with wounding and OS collected from larvae inoculated with *Enterobacter*. Gene expression was analyzed 24 h after treatment. Different letters indicate statistically significant differences between treatments (ANOVA, *P* < 0.05; followed by LSD test; *OsHI-LOX*, *F*_(2,15)_ = 166.7, *P* < 0.001; *OsAOS2*, *F*_(2,15)_ = 181, *P* < 0.001; *OsPAL1*, *F*_(2,15)_ = 60.68, *P* < 0.001; *OsPR-1a*, *F*_(2,15)_ = 73.35, *P* < 0.001; n = 6). CK, Untreated rice plants; OS+, OS collected from larvae inoculated with *Enterobacter*; OS-, OS collected from larvae inoculated with only MgCl_2_ solution

We further tested if the presence of *Enterobacter* and *Acinetobacter* would affect the performance of SSB larvae and cause cascade effect on defense responses in rice plants. Inoculation of both *Enterobacter* and *Acinetobacter* showed no effect on larval growth compared to that of axenic larvae inoculated with only buffer for 2 days of feeding (Fig. 6A; *F*_(2,100)_ = 1.118, *P* = 0.3309 > 0.05). However, larvae inoculated with either *Enterobacter* or *Acinetobacter* had higher penetration rate compared to that of larvae inoculated with only buffer (Fig. 6B; *F*_(2,6)_ = 19.06, *P* = 0.0025). In addition, both *Enterobacter* and *Acinetobacter* were detected in the damaged plant tissue infested with gnotobiotic larvae, while bacteria were not found on plants damaged by axenic larvae inoculated with only MgCl_2_ solution (Additional file 1: Fig. S1). OS collected from SSB larvae inoculated with *Enterobacter* suppressed LOX activities in rice stems (Fig. 7A; *F*_(2,42)_ = 20.43, *P* < 0.001), and larvae grew better on rice plants treated with OS collected from *Enterobacter*-inoculated axenic larvae (Fig. 7B; *F*_(2,113)_ = 54.83, *P* < 0.001). Moreover, the levels of *OsHI-LOX* and *OsAOS2* expression have been significantly suppressed, while up-regulated the expression of SA pathway-related Os*PAL1* and *OsPR-1a* genes have been observed in rice stems infested by OS collected from *Enterobacter*-inoculated larvae (Fig. 8). These data indicated that SSB larvae tended to secret detectable quantities of bacteria that suppressed defense response in rice plants, thus benefited the performance of SSB larvae.

## Discussion

In the current study, we demonstrated that SSB larvae secreted bacteria through their OS to mediate induced defense responses in rice plants. The levels of defense-related enzymes and genes associated with the JA signaling pathway increased in rice plants damaged by antibiotic-treated SSB larvae compared to non-AB treated larvae. In addition, bacteria from the OS of SSB caterpillars suppressed JA-mediated plant defenses, leading to enhanced performance of SSB larvae on rice plants. This is in agreement with previous studies indicating that the orally secreted bacteria of chewing insects are responsible for suppression of JA signaling by the activation of SA signaling in host plants (Chung et al. 2013; Acevedo et al. 2017).

Generally, rice plants infested by SSB larvae displayed higher anti-herbivore responses against insects (Li et al. 2020; Liu et al. 2021). RNAi-mediated silencing of *OsAOS2* involved in JA biosynthesis significantly reduced the resistance of rice plants to SSB infestation (Mei et al. 2006). Similarly, the transcripts of *OsHI-LOX* encoding chloroplast-localized type2 13-lipoxygenase were up-regulated in response to feeding by SSB larvae, and silencing *OsHI-LOX* involved in herbivore-induced JA biosynthesis increased the susceptibility of rice to SSB larvae (Zhou et al. 2009). However, SSB infestation also induced the up-regulation of genes such as *NPR1* (Nonexpressor of pathogenesis-related genes 1) and WRKY transcription factors, which have been shown to activate SA-responsive defense responses and inhibit JA-responsive defense responses (Eulgem and Somssich 2007). Interestingly, rice plants gradually changed from JA-responsive defense response to SA-responsive defense response after 3 h feeding by SSB larvae (Li et al. 2020). Consistent with previous findings, we found that OS collected from larvae without AB treatment triggered higher expression of *OsPAL1* and *OsPR-1a* genes compared to the treatment of OS collected from AB-treated larvae, while the expression levels of the JA-responsive *OsHI-LOX* and *OsAOS2* were lower in rice plants damaged by OS collected from non-AB treated larvae after 24 h. The phenylalanine ammonia-lyase gene (*PAL*) encodes a key enzyme involved in the biosynthesis of lignin, SA, and other phenylalanine-derived metabolites, which involve in resistance to both pathogens and pests (Tonnessen et al. 2015; You et al. 2020). For example, the gene *OsPAL1* can not only be triggered by *Magnaporthe oryzae* through SA signaling pathway (Duan et al. 2014), but also be up-regulated by the feeding of rice brown planthopper (*Nilaparvata lugens*) (He et al. 2020). The *OsPAL4* gene functioned similarly as *OsPAL1*, which was also associated with broad spectrum disease and insect resistance (Tonnessen et al. 2015; Lin et al. 2022). It deserved to investigate if *OsPAL4* has the same transcription pattern as *OsPAL1* in rice plants responding to the infestation of insects and microbes in the future. The SA inducible gene pathogenesis related gene *OsPR-1a* is treated as a marker gene in rice plants when interact with pathogens and insect pests (Agrawal et al. 2001; Feng et al. 2021; Satoh et al. 2009). Thus, SSB larvae may involve specific patterns to prevent rice plants from fully activating anti-herbivore defenses by up-regulation of SA-responsive defense responses.

Upon attack by herbivores, plants perceive herbivore-associated cues in OS and regulate biosynthesis of phytohormones and antiherbivore defense responses (Zhu et al. 2014; Acevedo et al. 2015). Herbivores can manipulate the perception and suppress plant induced defenses by exploiting effectors and/or their associated oral microbes. For example, an effector named HARP1 in the oral secretion of *Helicoverpa armigera* can directly interact with JASMONATE-ZIM-domain (JAZ) repressors to prevent the COI1-mediated JAZ degradation, thus blocking JA signaling-regulated antiherbivore defense responses (Chen et al. 2019). Furthermore, herbivorous insects tend to deposit OS containing certain quantities of bacteria to manipulate plant induced defense (Chung et al. 2013; Acevedo et al. 2017; Wang et al. 2018; Mason et al. 2019b; Sorokan et al. 2020). Similarly, we found that SSB larvae without AB treatment harbored more bacteria in their OS and deposited more bacteria around the feeding sites (Fig. 2). From the microbial community present in the OS of wild SSB larvae, we identified seven different bacteria genera. Among these isolates, *Enterobacter* and *Acinetobacter* were found to suppress JA-responsive defense when directly applied to rice plants. Our previous studies showed that *Enterobacter* and *Acinetobacter* isolated from the OS of *Leptinotarsa juncta* and the frass of *Leptinotarsa decemlineata* suppressed polyphenol oxidase activity in tomato plants and potato plants, respectively (Wang et al. 2016; Gao et al. 2022). In addition, changes in gene expression of maize inoculated with *Enterobacter* isolated from caterpillars were similar with maize gene profile change after insect feeding (Li et al. 2022). However, it remains to be further determined if other metabolites produced by SSB symbiotic bacteria activate the SA-signaling pathway to interfere with JA-regulated defenses.

Most recent studies about SSB symbiotic bacteria have focused on the role in detoxification of insecticides and adaptation to host plants or geographic locations (Zhang et al. 2013, 2022; Li et al. 2016; Lei et al. 2020; Zhong et al. 2021), while quite limited studies have reported about the role of SSB symbiotic bacteria in mediating the interaction between plants and insects. We concluded that SSB oral-secreted bacteria suppressed herbivore-induced defenses and enhanced insect performance on rice plants. Recognizing insect-associated bacteria as vital factors regulating the complex interaction between plants and insect herbivores may contribute to our understanding of how SSB larvae adapt to rice plants in a wide range of agroecosystems.

## Supplementary Information

**Additional file 1: Table S1.** The information of primers used in this study. **Figure S1.** Detection of *Enterobacter* and *Acinetobacter* on the feeding sites of rice stems damaged by gnotobiotic larvae. Larvae inoculated with either *Enterobacter* or *Acinetobacter* were allowed to feed on rice plants for about one hour. Then damaged sites of rice stems (100 mg) were collected, and cultured in 5 mL of 2YT media overnight. Tissues collected from undamaged plants were also cultured in 2YT media. DNA was extracted from those cultures, and the specific primer of both *Enterobacter* and *Acinetobacter* (Table S1) were used for detection by PCR. M: DNA size marker (2000 bp), lane 1 and 4: DNA isolated from undamaged rice stems; lane 2: DNA isolated from *Enterobacter*; lane 3: DNA isolated from rice stems damaged by *Enterobacter*-inoculated SSB larvae; lane 5: DNA isolated from *Acinetobacter*; lane 6: DNA isolated from rice stems damaged by *Acinetobacter* -inoculated SSB larvae.

## Data Availability

All data supporting the findings of this study are available from the corresponding author on request.
