# Supplementary material for: Rice Defense Responses Orchestrated by Oral Bacteria of the Rice Striped Stem Borer, Chilo suppressalis
Source: Rice (N Y). 2023 Jan 9;16:1. doi: 10.1186/s12284-022-00617-w (PMC9829949; doi:10.1186/s12284-022-00617-w)
Supplement: Supplementary file 1 — Additional file 1: Table S1. The information of primers used in this study. Figure S1. Detection of Enterobacter and Acinetobacter on the feeding sites of rice stems damaged by gnotobiotic larvae. Larvae inoculated with either Enterobacter or Acinetobacter were allowed to feed on rice plants for about one hour. Then damaged sites of rice stems (100 mg) were collected, and cultured in 5 mL of 2YT media overnight. Tissues collected from undamaged plants were also cultured in 2YT media. DNA was extracted from those cultures, and the specific primer of both Enterobacter and Acinetobacter (Table S1) were used for detection by PCR. M: DNA size marker (2000 bp), lane 1 and 4: DNA isolated from undamaged rice stems; lane 2: DNA isolated from Enterobacter; lane 3: DNA isolated from rice stems damaged by Enterobacter-inoculated SSB larvae; lane 5: DNA isolated from Acinetobacter; lane 6: DNA isolated from rice stems damaged by Acinetobacter -inoculated SSB larvae. [file 12284_2022_617_MOESM1_ESM.doc]

**Table S1 The information of primers used in this study**

| **Gene** | **5'-3'** | **Reference**  **(GenBank Accession)** |
| --- | --- | --- |
| *OsPAL1* | F-CACAAGCTGAAGCACCACCC | (Yang et al. 2022) |
|  | R-GAGTTCACGTCCTGGTTGTG |  |
| *OsPR-1a* | F-GTGGACCCGCACAACGCG | (Yang et al. 2022) |
|  | R-GCGATCGCCGTCGAGTC |  |
| *OsActin* | F-ACTGTCCCCATCTA TGAAGGA | (Yang et al. 2022) |
|  | R-CTGCTGGAATGTGCTGAGAGA |  |
| *OsHI-LOX* | F-ACCCTAAGTCGGAGACGAGG | (Zhou et al. 2009) |
|  | R-GTCTTGCGGAGGAAGTAGTCC |  |
| *OsAOS2* | F-CAAGAAGGGGGAGATGCTGTT | (Mei et al. 2006) |
|  | R-TCTCGCCCATTCGACCAGTA |  |
| *Enterobacter* | F-ATACCCTGGTAGTCCACGCC | MZ424726 |
|  | R-TCGCTTCTCTTTGTATGCGC |  |
| *Acinetobacter* | F-ACTGGTTGGCTAGAGTGTGG | MZ424720 |
|  | R-CCAACATCTCACGACACGAG |  |

**References**

Mei C, Qi M, Sheng G, Yang Y (2006) Inducible overexpression of a rice allene oxide synthase gene increases the endogenous jasmonic acid level, PR gene expression, and host resistance to fungal infection. Mol Plant-Microbe Interact 19:1127–1137. https://doi.org/10.1094/MPMI-19-1127

Yang X, Gu X, Ding J, et al (2022) Gene expression analysis of resistant and susceptible rice cultivars to sheath blight after inoculation with Rhizoctonia solani. BMC Genomics 1–16. https://doi.org/10.1186/s12864-022-08524-6

Zhou G, Qi J, Ren N, et al (2009) Silencing *OsHI-LOX* makes rice more susceptible to chewing herbivores, but enhances resistance to a phloem feeder. Plant J 60:638–648. https://doi.org/10.1111/j.1365-313X.2009.03988.x

**Fig. S1 Detection of *Enterobacter* and *Acinetobacter* on the feeding sites of rice stems damaged by gnotobiotic larvae.** Larvae inoculated with either *Enterobacter* or *Acinetobacter* were allowed to feed on rice plants for about one hour. Then damaged sites of rice stems (100 mg) were collected, and cultured in 5 mL of 2YT media overnight. Tissues collected from undamaged plants were also cultured in 2YT media. DNA was extracted from those cultures, and the specific primer of both *Enterobacter* and *Acinetobacter* (Table S1) were used for detection by PCR. M: DNA size marker (2000 bp), lane 1 and 4: DNA isolated from undamaged rice stems; lane 2: DNA isolated from *Enterobacter*; lane 3: DNA isolated from rice stems damaged by *Enterobacter*-inoculated SSB larvae; lane 5: DNA isolated from *Acinetobacter*; lane 6: DNA isolated from rice stems damaged by *Acinetobacter* -inoculated SSB larvae.

**
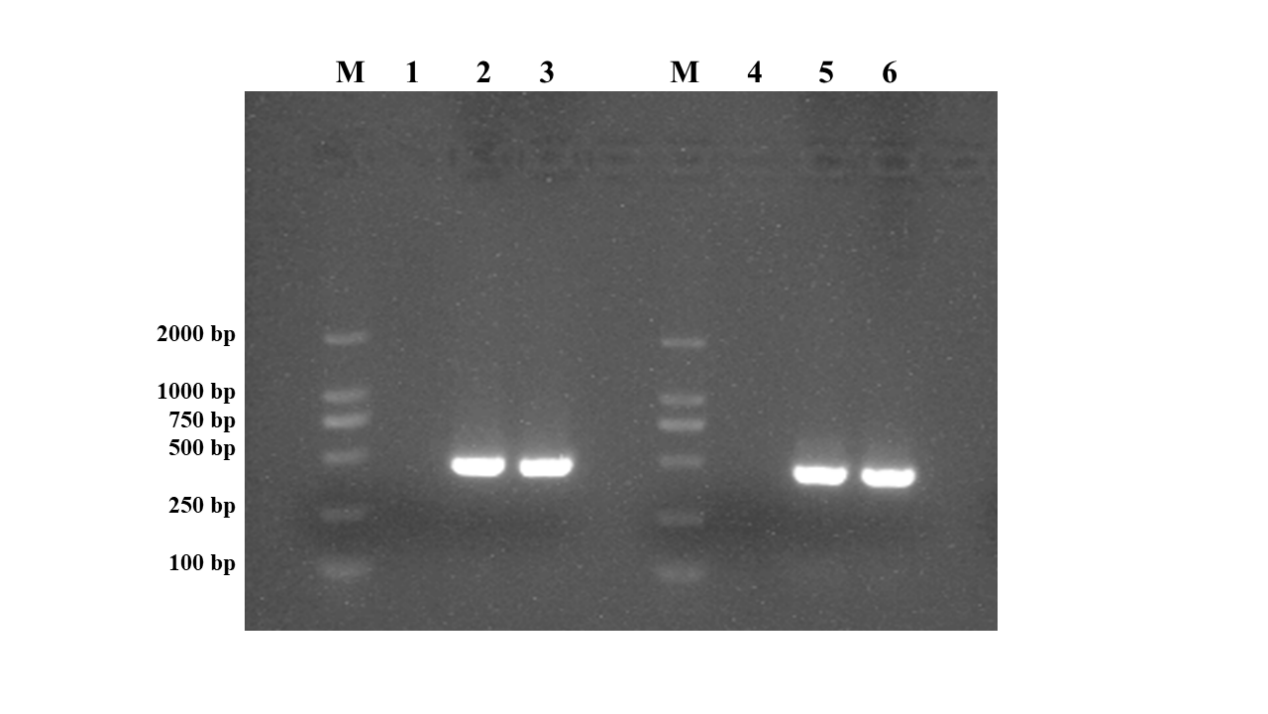
**
